# An In Vitro–In Vivo Simulation Approach for the Prediction of Bioequivalence

**DOI:** 10.3390/ma14030555

**Published:** 2021-01-24

**Authors:** Marilena Vlachou, Vangelis Karalis

**Affiliations:** Department of Pharmacy, School of Health Sciences, National and Kapodistrian University of Athens, 15784 Athens, Greece; vlachou@pharm.uoa.gr

**Keywords:** modeling and simulation, drug release, bioequivalence, in vitro dissolution

## Abstract

The aim of this study was to develop a new in vitro–in vivo simulation (IVIVS) approach in order to predict the outcome of a bioequivalence study. The predictability of the IVIVS procedure was evaluated through its application in the development process of a new generic product of amlodipine/irbesartan/hydrochlorothiazide. The developed IVIVS methodology is composed of three parts: (a) mathematical description of in vitro dissolution profiles, (b) mathematical description of in vivo kinetics, and (c) development of joint in vitro–in vivo simulations. The entire programming was done in MATLAB^®^ and all created scripts were validated through other software. The IVIVS approach can be implemented for any number of subjects, clinical design, variability and can be repeated for thousands of times using Monte Carlo techniques. The probability of success of each scenario is recorded and finally, an overall assessment is made in order to select the most suitable batch. Alternatively, if the IVIVS shows reduced probability of BE success, the R&D department is advised to reformulate the product. In this study, the IVIVS approach predicted successfully the BE outcome of the three drugs. During the development of generics, the IVIVS approach can save time and expenses.

## 1. Introduction

The aim of bioequivalence (BE) testing is to assess the in vivo equivalence between two medicinal products of the same active substance [1,2]. In order to accomplish this task, a pharmacokinetic comparison is made between the two medicinal products, namely, the one under comparison (the generic or Test (T)) against the originator’s pharmaceutical product (Reference, R). Following a specific statistical assessment, if the T formulation is proved equivalent to the R, in terms of pharmacokinetics, then it is also considered therapeutically equivalent. In other words, BE testing is performed in the ground of pharmacokinetics, but the proof of equivalence is extrapolated to the area of therapeutics.

Since, in BE studies the two pharmaceutical products have the same active substance, the disposition will be the same and what differentiates them can mainly be attributed to the absorption process due to the impact of the excipients and formulation. Thus, it was recognized that understanding the in vitro drug release performance, represents a key factor in the generic pharmaceutical development [3]. It is very important in the research and development (R&D) process, the early selection and decision on the formulation strategies, which will lead to the development of a product with the highest possibility of showing bioequivalence [4].

During the last years, in vitro dissolution studies have become the most widely utilized tool in the development process of generics. The goal of dissolution studies is to assure quality and to serve as a surrogate for drug bioavailability [3]. In particularly, for drugs with low solubility, where dissolution represents the rate limiting step in absorption, comparative dissolution studies provide useful insights for the in vivo bioequivalence [5]. During the past years, it was acknowledged that mathematical and computational tools can be used to consistently correlate dissolution testing with in vivo data and thus the concept of in vitro–in vivo correlations (IVIVC) was developed [3,6,7,8]. IVIVC refer to predictive mathematical models describing the relationship between a characteristic related to dissolution properties (e.g., extent or rate of dissolution) and another in vivo property (e.g., plasma concentration) of the dosage form.

In principle, the goal of IVIVC is to reduce the cost and time of drug development duration, ensure batch to batch quality, assist the quality control for scale-up and possible post-approval changes, use IVIVC predictions as a surrogate for in vivo bioavailability, and in a subsequent step to support biowaivers [7]. In this vein, IVIVC have been developed not only for chemical drugs, but also for monoclonal antibodies in order to establish correlations between the in vitro efficacy and the in vivo effect of monoclonal antibody conjugates [9]. For these reasons, the use of IVIVC studies is greatly acknowledged by pharmaceutical companies developing generics and invest on them, even though their use appears to be underrated in submissions for regulatory approval [10].

One important issue in dissolution testing is the discriminatory ability of in vitro media. Towards this issue several attempts have been made to change appropriately the dissolution conditions in order to reflect better the in vivo performance of the drug and optimize predictability. In this context, several efforts have been made including the use of dissolution-absorption or dissolution-permeation simulating systems, artificial duodenum-stomach models, Caco-2 cells combined with dissolution apparatuses, and biphasic dissolution approaches [11]. Finally, in the recent years the development of biorelevant media, as input into physiologically based absorption modeling, has been proved a useful tool for the prediction of in vivo performance [12,13]. In the same vein, biopredictive media have been assessed in conjunction with IVIVC approaches [14].

In silico mathematical modeling and simulation represents a substantial part of the research and development in pharmaceutical industry aiming to optimize the design of dosage forms [15]. Computer simulations have been used in many ways such as physiologically based pharmacokinetic (PBPK) models, clinical trial simulations, software tools that predict the systemic pharmacokinetics of certain drugs (e.g., inhalers), and mathematical models to predict bioequivalence [16,17,18,19,20,21,22]. In case of PBPK modeling, certain software platforms are widely used in pharmaceutical industry to simulate drug absorption through a variety of administration routes (such as oral, inhalation, subcutaneous, ocular) and predict pharmacokinetics and pharmacodynamics in animals and humans [16,17,18,19]. These PBPK software tools are capable of consistently predicting the in vivo performance of formulations and rationalize product development. Additionally, other software platforms simulate several aspects of clinical trials (e.g., variability, sample size, dosage regimens, pathological status) and allow predicting the probability of success [18]. Other software tools have revolutionized pharmacokinetic/pharmacodynamic modeling and simulation through the application of non-linear mixed effects models [23,24,25]. Besides, semi-mechanistic simulation tools have been developed for specific needs, as for example the prediction of systemic kinetics of inhaled corticosteroids (e.g., budenoside, fluticasone propionate, mometasone furoate, flunisolide) delivered via dry powder inhalers [20,21].

In addition, computer simulations have been applied in bioequivalence in order to address issues like the use of parent drugs or metabolite, the statistical framework, and the development of scaled BE limits [26,27,28,29,30,31,32,33,34,35,36,37,38,39]. In addition, modeling and simulation approaches have appeared in the literature using in vitro dissolution data for the prediction of in vivo pharmacokinetic profiles, such as assessing the impact of dissolution variability on in vivo pharmacokinetic profile or to evaluate BE of fluconazole capsules [40,41].

Today, computational methods are widely used in pharmaceutical development and there is a continuous need to expand the translation of in vitro data into in vivo performance, in order to assist R&D and provide reliable predictions [42]. In this context, the EMA has established a modeling and simulation working party aiming to offer scientific support to other EMA’s committees regarding modeling aspects relevant to medicines [43]. Additionally, the US-FDA has already issued guidelines providing suggestions for the application of PBPK modeling and population pharmacokinetic models [44,45]. Finally, the recent communication from the European Parliament, regarding pharmaceutical strategy in Europe, supports the use of in silico techniques and virtual approaches for reducing costs and decrease development times [46].

The aim of this study is to develop a new in vitro–in vivo simulation (IVIVS) approach in order to predict the in vivo outcome of a bioequivalence study, relying on in vitro dissolution data and literature information. An early version of the IVIVS approach has been presented for the prediction of BE in case of two losartan studies [47]. In this study, we describe the IVIVS procedure and evaluate its predictability through its application during the R&D of a triple combination oral immediate release tablet of amlodipine/irbesartan/hydrochlorothiazide (10/300/12.5 mg).

## 2. Materials and Methods

### 2.1. The IVIVS Methodology

**Figure 1:**
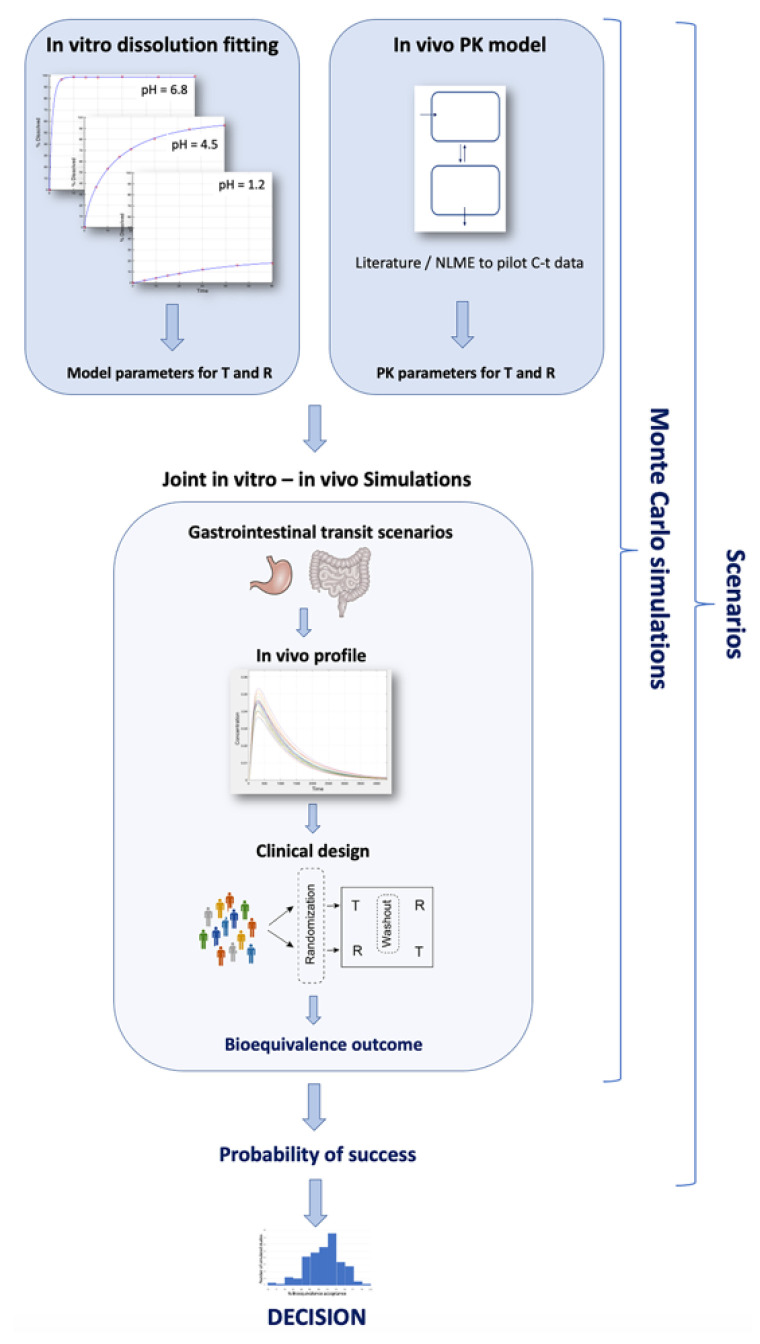
A schematic representation of the in vitro–in vivo simulation (IVIVS) approach. The first step refers to fittings of the in vitro dissolution data. The pharmacokinetic model is built using literature information from non-linear mixed effect (NLME) modeling or other pharmacokinetic data. Then, joint in vitro–in vivo simulations are used to generate virtual subjects, gastrointestinal transit scenarios, and a clinical design. The entire procedure is repeated for thousands of times using Monte Carlo simulations. All previous steps are performed for several scenarios like different sample size, sampling scheme, and variabilities.

The developed IVIVS methodology can be summarized in the following three steps: (a) Mathematical description of in vitro dissolution profiles, (b) Mathematical description of in vivo pharmacokinetics, and (c) Development of joint in vitro–in vivo simulations (Figure 1).

#### 2.1.1. Step 1: Mathematical Description of the In Vitro Dissolution Profiles

In a first step, mathematical models (e.g., first-order, zero-order, Weibull, logistic, Peppas model) are used to describe the individual dissolution profiles at the three buffers imposed by the guidelines, namely, pH of 1.2, 4.5, and 6.8. (Figure 1). The pH values of 1.2, 4.5, and 6.8 refer to the conditions of the simulated fasted stomach, fed stomach, and small intestine, respectively. The same procedure is followed for both the T and R formulations (e.g., using 12 units of each formulation), since during the development of a generic product, dissolution data are available for both formulations as a part of the R&D routine. The choice of the mathematical model is based on the fittings results and the derived statistical criteria like Akaike Information Criterion (AIC) and Bayesian Information Crietrion (BIC) for each fitting.

It should be stated that in the IVIVS approach these models are only used to describe the dissolution longitudinal data and not for understanding or providing answers for the theory behind drug release. Dissolution fittings can be performed to the dissolution data of each vessel separately, or otherwise a non-linear mixed effect method can be applied simultaneously to all data of each pH condition. Thus, after this step, the individual and average dissolution model parameter estimates are obtained.

#### 2.1.2. Step 2: Mathematical Description of In Vivo Pharmacokinetics

The second stage in the IVIVS methodology (Figure 1), refers to the establishment of a pharmacokinetic model to describe in vivo kinetics. This can usually be accomplished by identifying the relevant pharmacokinetic information from the literature. Then using the pharmacokinetic parameter values reported in the literature, simulations can be made for the in vivo behavior. Alternatively, if results from a pilot pharmacokinetic study are available, then non-linear mixed effect modeling can be applied for estimating the pharmacokinetic model parameters.

**Figure 2:**
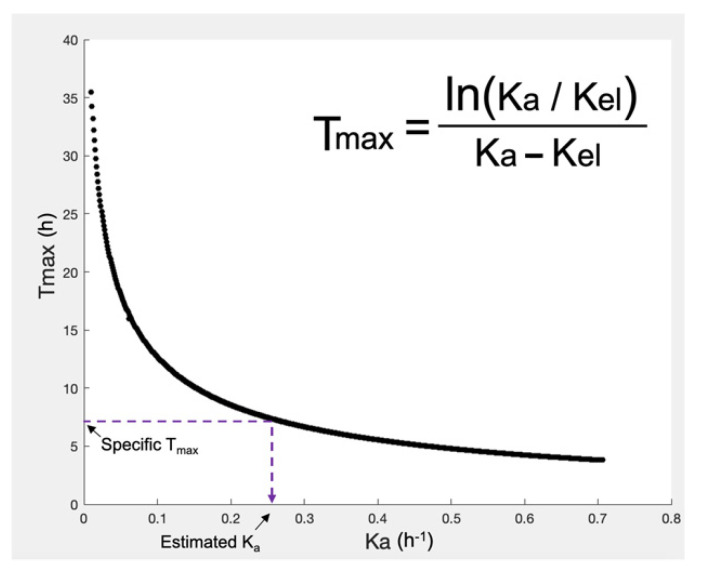
A graphical representation of the computational method used to calculate absorption rate constant (Ka) from Tmax (i.e., the time at which the maximum concentration is observed) and elimination rate constant (Kel). The equation shown in the insert cannot be solved in terms of Ka. Thus, a code was created in MATLAB^®^ allowing the numerical solution in terms of Ka, as follows: since, the Kel is known then several Ka values are iteratively used to estimate Tmax. The Ka value leading to the correct Tmax represents the solution.

Since, the in vivo disposition of a drug is not influenced by the formulation characteristics, the pharmacokinetic estimates (e.g., clearance, apparent volumes of distributions) can be considered the same for both the T and R product. When the required information is not directly available from the literature, extra calculations can be made. For example, when elimination rate constant (Kel) is not reported, it can be calculated from clearance and volume of distribution, using the typical pharmacokinetic mathematical formulas. Additionally, when the absorption rate constant (Ka) values are not known, they can be estimated from the reported Tmax (i.e., the time at which the maximum plasma concentration (Cmax) is observed) and the Kel estimates. In this study, in order to accomplish this task, a computational method was developed which provided the numerical solution of Ka from Tmax and Kel values (Figure 2). Alternatively, estimations can be made using results from reported bioequivalence (BE) analysis, since from the reported Cmax, Tmax, the 90% confidence intervals, almost all necessary pharmacokinetic parameters can be calculated.

In this study, the in vivo pharmacokinetic parameters for the three drugs (amlodipine, irbesartan, hydrochlorthiazide) were taken from the literature. For amlodipine, a one-compartment model with first order absorption and elimination was used, while the kinetics of irbesartan and hydrochlorthiazide kinetics were described by a two-compartment model with lag-time in absorption and first-order kinetic transfers [48,49,50,51,52].

#### 2.1.3. Step 3: In Vitro–In Vivo Simulations

**General**

The third step of the IVIVS approach refers to the application of joint in vitro–in vivo simulations using the mathematical models and parameters obtained from the previously two steps. In all cases, mathematical description is implemented through systems of ordinary differential equations. 

Initially, virtual subjects are created assuming a statistical distribution (usually lognormal distribution), for the values of each pharmacokinetic parameter, using the mean and variability estimates computed in the previous steps of the analysis. Any number of subjects (N) can be simulated and these virtual subjects can randomly be split into the two groups of the study, receiving either the T or R formulation. Between- and within-subject variability is added, as well as a residual error model (proportional, additive, combined) is applied. For the purposes of the current analysis, a proportional error model was used in the simulations for all three compounds (amlodipine, irbesartan, and hydrochlorthiazide). 

**Gastrointestinal transit scenarios**

In case of step 1, the estimated dissolution parameters refer to specific regions of the gastrointestinal (GI) tract. Thus, the condition of a fasted stomach is considered to be reflected on a medium with pH 1.2, while fed stomach conditions refer to pH 4.5. The simulated small intestine is reflected on the buffer with a pH of 6.8.

Since different dissolution kinetics can be observed in each pH, the residence of the drug in each simulated GI location is important for the in vivo performance. In this study, the selection of the mean residence time in each GI section was based on literature values [53]. In order to have more reliable simulation of the GI transit, several scenarios encompassing different mean residence time in each region (stomach, small intestine, large intestine) were investigated (Table 1). Aiming to place emphasis on gastric emptying, special focus was placed on transit scenarios with short residence in stomach.

Plausibly, since at each segment of the gastrointestinal system (stomach, small- and large-intestine) exist different conditions (e.g., pH, permeability etc.), this has an impact on the absorption characteristics of the drug. For example, in the stomach the absorption can be negligible or low, depending on the drug. Thus, residence for a long time in the stomach (as in case of fed conditions) will result in a delay in the appearance of drug in plasma. Additionally, in fed conditions, the dissolution kinetics used in the IVIVS model, would utilize the model parameter values resulting from the in vitro dissolution at pH 4.5, which can be different from that at 1.2. Therefore, using different residence times at each part of the gastrointestinal system can lead to different results. In the same context, differences observed in vitro are translated into in vivo performances and these differences can be amplified or shrunk by adjusting appropriately (i.e., physiologically sound) model parameters (such as residence times, variabilities etc.).

**Bioequivalence estimation**

For each simulated concentration—time (C-t) profile, a specific sampling scheme is simulated. Based on the sampling schedule, for each one of the N virtual subjects, the selected C-t data serve for the calculation of the area under curve (AUC) and Cmax using the typical non-compartmental approach. Besides, several sampling schemes (e.g., sparse, typical, dense) can be examined in order to select the most appropriate, namely the one that balances statistical power of the study and cost. The AUC and Cmax for all subjects are appropriately adapted to a virtual clinical design (e.g., a 2 × 2 crossover design was assumed in this study). Depending on the clinical design the relevant statistical assessment for BE is applied, as imposed by the regulatory authorities [1,2]. In case of the typical 2 × 2 design, the latter includes logarithmic transformation of AUC and Cmax, application of ANOVA in order to estimate the residual error, and then the 90% confidence interval is calculated around the geometric mean ratio of the BE measure (i.e., AUC and Cmax). If the 90% confidence interval lies within the predefined limits of acceptance (usually 80–125%) then BE is declared.

**Monte Carlo simulations—Probability of success**

The abovementioned procedure, of simulating a virtual BE study, is repeated many times (thousands/millions) using Monte Carlo techniques and the success or fail of each study is recorded. After performing all repetitions, the % probability of BE acceptance can be obtained.

**Figure 3:**
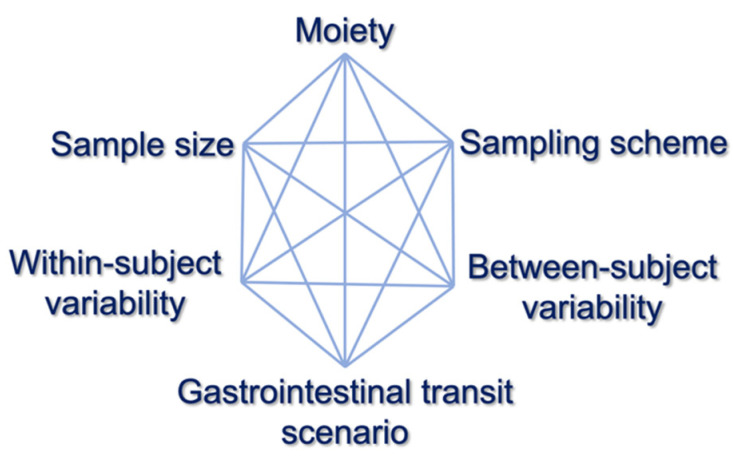
Factors affecting the outcome of a bioequivalence study. These factors include sampling scheme, between- and within-subject variability of the virtual subjects, gastrointestinal transit scenario (e.g., slow or fast gastric emptying), sample size, and moiety (e.g., parent drug or metabolite).

In addition, the entire procedure is repeated several times in order to investigate many different factors that may affect the outcome of the BE study (Figure 3), namely, between/within-subject variability, GI transit time scenario, sample size, and sampling scheme. Thus, these probabilities of successes are obtained which can be plotted into a histogram, to allow a pictorial view of the overall performance.

During the R&D process, the abovementioned procedure can be applied to all batches and the relevant % probabilities of BE acceptance will be obtained. Judging from these results, the batch with the overall highest probability of BE acceptance will be the most preferable. Thus, the R&D department can decide on the fate of the development program; if many formulation batches have been developed, the R&D group can choose the appropriate batch for the pivotal study. In case when only batch has been developed, then based on the IVIVS results, they can choose whether it is safe to continue with this formulation or it is preferable to re-formulate and apply again the IVIVS to assess the new possibility of BE acceptance of the re-formulated product.

### 2.2. Application to Actual Data: The Cases of Amlodipine, Irbesartan, Hydrochlorthiazide

In order to evaluate the predictability of this methodology, the IVIVS approach was applied in the development process of an oral immediate release tablet consisting of the triple combination amlodipine/irbesartan/hydrochlorothiazide (10/300/12.5 mg) of Elpen SA Pharmaceutical Industry. The IVIVS was applied to several batches of the new tablet which were compared with the two originator’s products (Istin^®^ 10 mg/Pfizer, CoAprovel^®^ 300/12.5 mg/Sanofi-Aventis).

The analysis followed the typical procedure presented in Figure 1. The aim of this application was to identify the batch that leads to the highest overall probability of declaring BE concomitantly for the three active substances. Based on the findings of the IVIVS analysis, one batch was selected for the subsequent pivotal BE study.

The factors assessed in the study of the triple tablet referred to five sample sizes (N = 12, 18, 24, 30, 36), five GI transit scenarios (see Table 1), three sampling schemes (Table 2), three levels of between-subject variability (5%, 10%, and 20%), and three levels of within-subject variability (5%, 10%, and 20%). 

Assuming a latin-square design of the experiment, 675 scenarios (i.e., 5 × 3 × 5 × 3 × 3 = 675) should had been examined for each active substance; thus, 675 × 3 = 2025 in total. Due to this high number of scenarios, a selection of the most probable scenarios was made in order to reduce the computational load. Thus, the number of evaluated scenarios was finally equal to 240 (80 for each compound) for each batch. Under each scenario, a number of 10,000 simulated BE studies was performed. In this study, only the results of the final selected batch will be presented. 

The list of scenarios investigated in the analysis of the amlodipine/irbesartan/hydrochlorothiazide tablet are listed in (Table 3).

### 2.3. Validation

In this study, the entire computational work was implemented in MATLAB^®^ version R2018b (Mathworks Inc.) using a graphical user interface (GUI). The validation of the IVIVS approach was performed in two ways: (a) by comparing the results of each part of the code with other software and the literature, (b) by assessing the predictive ability using the actual data from the bioequivalence study of amlodipine, irbesartan, and hydrochlorthiazide. Initially, all scripts created for the application of IVIVS were validated, step-by-step, during the development of the code. All critical parts of the code have been extensively utilized in the past for research in the field BE modeling [54]. In addition, the validation of the IVIVS code was made though comparison of the results with other established software. In the IVIVS code, the numerical solution of the system of ordinary differential equations describing in vitro dissolution and in vivo kinetics was implemented though the use of the *ode45* function. The results of these simulations were compared with the those of Simulx (Monolix^®^ 2020R1, Lixoft). The calculated pharmacokinetic parameters (AUC, Cmax, Tmax) of the IVIVS were compared with the pharmacokinetic estimates from the PKanalix tool (Monolix^®^ 2020R1, Lixoft). Finally, bioequivalence assessment was compared with the results of WinNonlin^®^ v.5.0.1/Pharsight Corp., Menlo Park, CA). Additionally, the predictive ability of the IVIVS approach was tested in the bioequivalence study conducted by Elpen SA for amlodipine, irbesartan, and hydrochlorthiazide. Since, three compounds were assessed, BE assessment was performed independently for each one of them.

## 3. Results

In this study, a new in vitro–in vivo simulation approach has been developed which utilizes in vitro dissolution data in order to make predictions for the probability of BE acceptance (Figure 1). It is a three-step procedure that can be split into the steps of: in vitro dissolution fitting, establishment of a pharmacokinetic model, and lastly the application of joint in vitro–in vivo simulations. In order to evaluate the predictive ability, the IVIVS approach was applied to the development process of a new anti-hypertensive generic product containing three active compounds (amlodipine, irbesartan, hydrochlorthiazide).

Initially, fittings to in vitro dissolution data were separately performed for each one dissolution curve of the 12 vessels for the T and the R formulations, at the three buffers (1.2, 4.5, and 6.8) for all three drugs. Several mathematical models (like first-order, logistic, Weibull, Peppas) were tested to the dissolution data and the simplest one offering the best results in terms of statistical criteria (e.g., AIC, BIC) was selected. Then, from the individual parameter estimates, the average dissolution parameter values were calculated.

**Figure 4:**
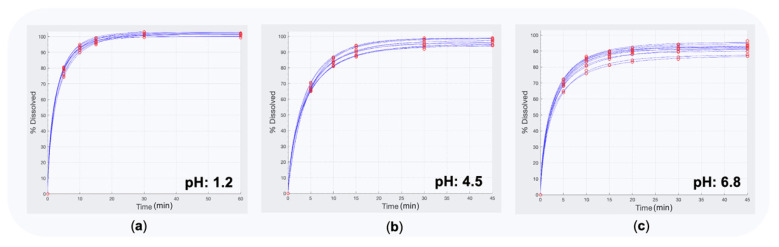
In vitro dissolution fittings for hydrochlorthiazide data performed in the first step of the IVIVS approach. In these fittings, first-order kinetics is applied to describe dissolution of hydrochlorthiazide at the three buffers: (**a**) pH 1.2, (**b**) pH 4.5, and (**c**) pH 6.8.

Table 4 lists the mean model parameter estimates for the T formulation at the three buffers for the three drugs. Similar estimations were made for the R product. The first-order model (with parameters the dissolution rate constant (Kd) and the amount dissolved (m0)) was the simplest model found that described nicely the dissolution data of the T and R products. Some indicative plots of the in vitro fittings of the Test formulation regarding hydrochlorthiazide are depicted in Figure 4.

The next step in the analysis was the determination of the appropriate pharmacokinetic model for each drug, as well as its parameter values. Initially, a literature search was performed to identify the pharmacokinetic model and the parameter estimates for the three drugs. In case of amlodipine and irbesartan, relevant information was reported in the bibliography and utilized in the simulations [48,49,50]. For hydrochlorthiazide, the necessary pharmacokinetic data were obtained from two relevant literature sources [51,52]. In this case, the Ka estimate was calculated from the reported Tmax and Kel values using a computational method for the numerical solution of Ka (Figure 2).

**Figure 5:**
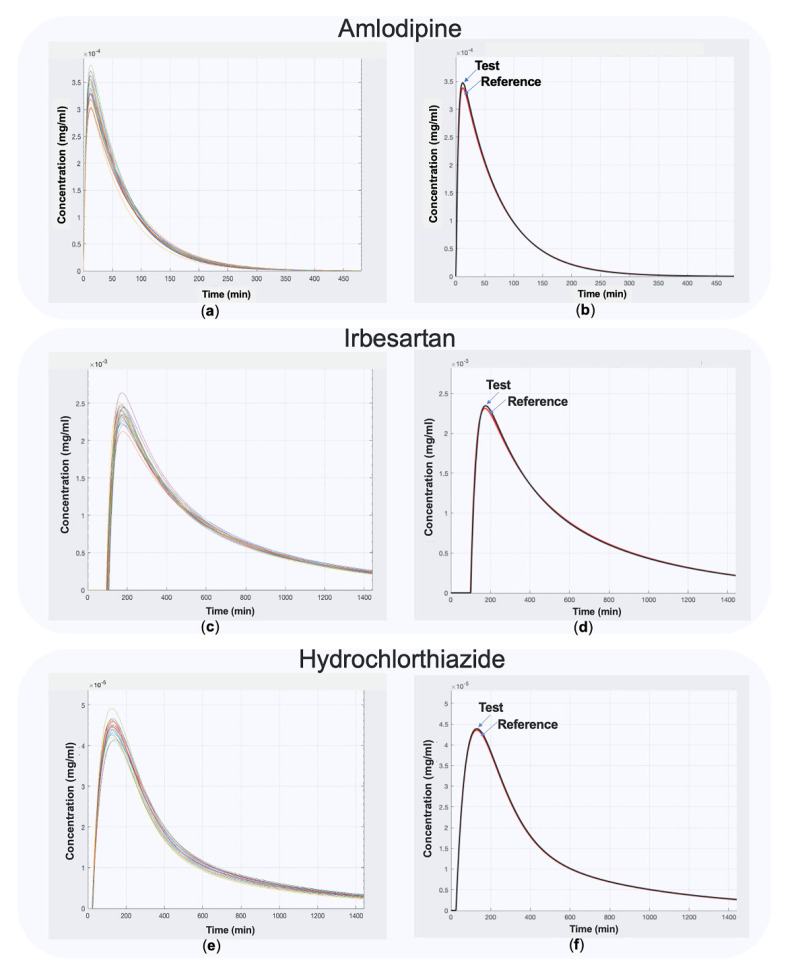
Simulated pharmacokinetic C-t profiles for amlodipine, irbesartan, and hydrochlorthiazide using the IVIVS approach. In these simulations, 24 subjects were generated assuming a between-subject variability of 5%. The left column plots (**a**,**c**,**e**) refer to individual C-t profiles of amlodipine, irbesartan, and hydrochlorthiazide, respectively. The right column plots (**b**,**d**,**f**) represent the average performance of the Test and Reference formulations of amlodipine, irbesartan, and hydrochlorthiazide, respectively.

**Figure A1:**
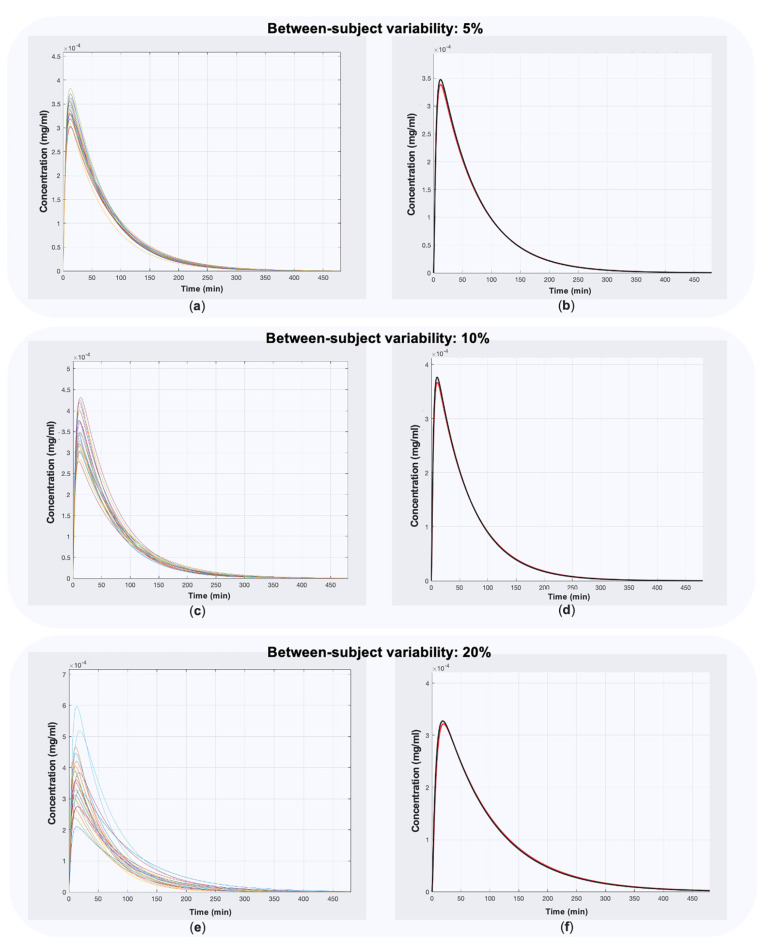
Simulated C-t profiles for amlodipine using the IVIVS approach. In these simulations, 24 subjects were generated assuming between-subject variability of 5% (**a**,**b**), 10% (**c**,**d**), and 20% (**e**,**f**). The left column plots (**a**,**c**,**e**) refer to the individual C-t profiles of the 24 virtual subjects. The right column plots (**b**,**d**,**f**) represent the average performance of the Test and Reference formulations.

**Figure A2:**
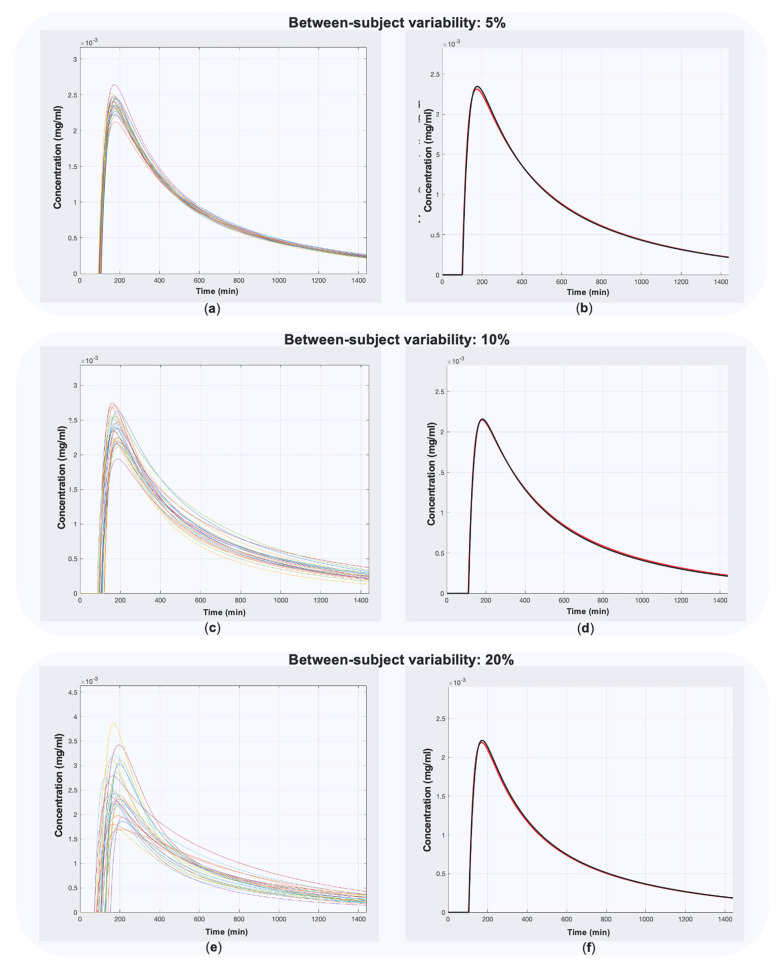
Simulated C-t profiles for irbesartan using the IVIVS approach. In these simulations, 24 subjects were generated assuming between-subject variability of 5% (**a**,**b**), 10% (**c**,**d**), and 20% (**e**,**f**). The left column plots (**a**,**c**,**e**) refer to the individual C-t profiles of the 24 virtual subjects. The right column plots (**b**,**d**,**f**) represent the average performance of the Test and Reference formulations.

**Figure A3:**
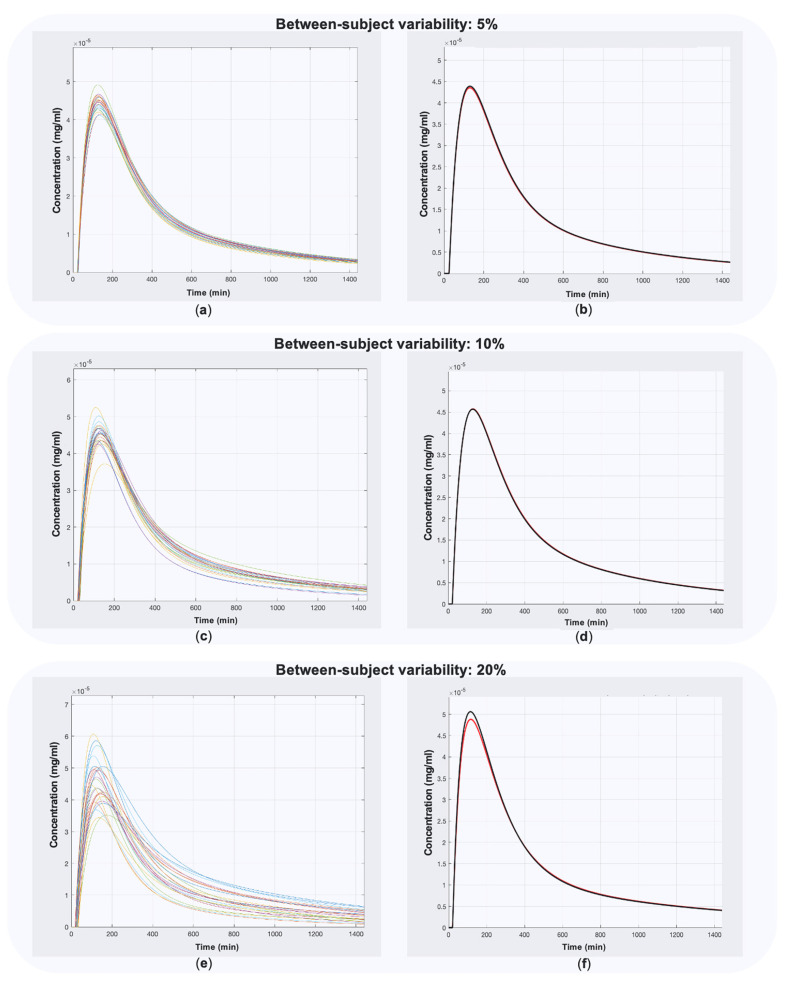
Simulated C-t profiles for hydrochlorthiazide using the IVIVS approach. In these simulations, 24 subjects were generated assuming between-subject variability of 5% (**a**,**b**), 10% (**c**,**d**), and 20% (**e**,**f**). The left column plots (**a**,**c**,**e**) refer to the individual C-t profiles of the 24 virtual subjects. The right column plots (**b**,**d**,**f**) represent the average performance of the Test and Reference formulations.

After the determination of the in vitro and in vivo model parameters, the next step of the procedure was the development of the in vitro–in vivo simulations for the three drugs (Figure 1). An indicative example with N = 24 randomly generated subjects is shown in Figure 5. Additionally, as between subject variability increases, a dispersion of the C-t curves is observed in all spaghetti plots for the three drugs (Figure A1, Figure A2 and Figure A3 in Appendix A). The figures shown in the Appendix A are only for illustrative purposes, to indicate how the dispersion of the C-t curves rises with the increase of between-subject variability (applied to all pharmacokinetic parameters). In case of the IVIVS application to the true example of the three drugs several sample sizes, variability levels, and other factors (according to Table 3) were analyzed. In the same vein, a representative example of a system of ordinary equations is shown in Table A1 of the Appendix A.

**Figure 6:**
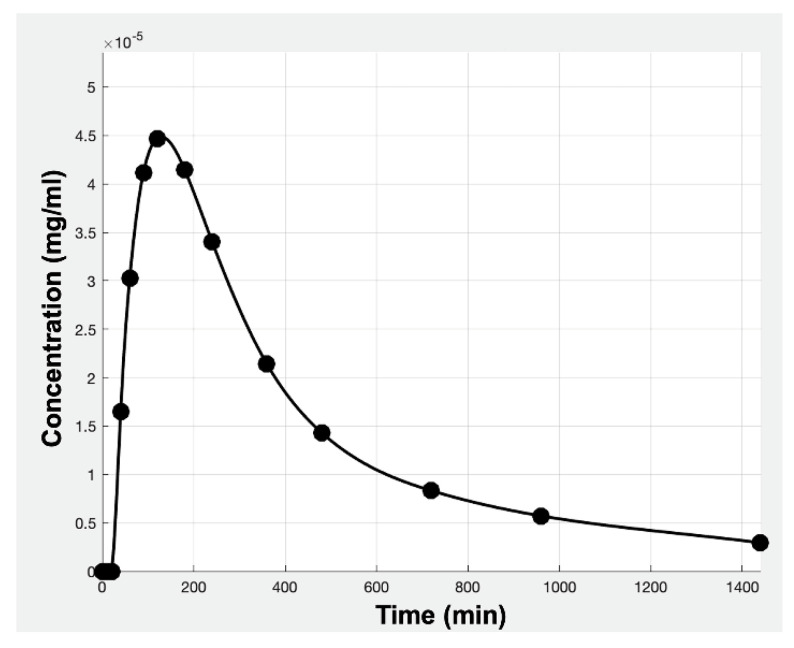
A simulated C-t profile of hydrochlorthiazide for a virtual subject. Dots refer to the selected concentration points from the sampling scheme.

After applying a specific sampling scheme to the simulated C-t data, certain (C, t) pairs were selected (Figure 6) and were further used for the calculation of AUC and Cmax. 

**Figure 7:**
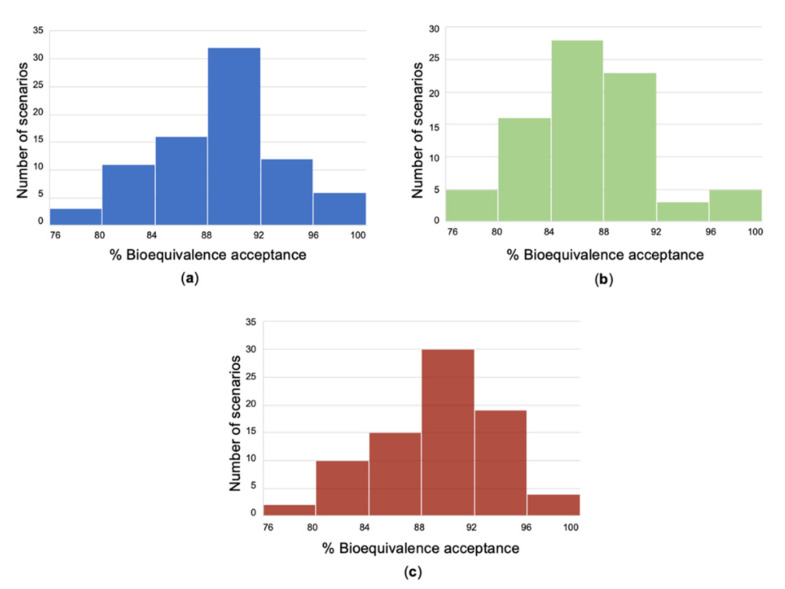
Histograms showing the distribution of % probability of bioequivalence success. Each histogram refers to the 80 scenarios investigated for finally selected (i.e., best) batches amlodipine (**a**), irbesartan (**b**), and hydrochlorthiazide (**c**).

Then, BE assessment took place and if the 90% CI lied within the pre-defined limits of 80–125%, the simulated BE study was considered successful. Using Monte Carlo simulations, the same procedure was repeated 10,000 times for each scenario and the number of successes or failures was recorded. This methodology was performed for several scenarios (Figure 3, Table 3), namely, situations with different combinations of sample size, variability of parameters, sampling schemes, and GI transit times. For every drug, a number of 80 scenarios was evaluated and the percent probability of BE acceptance was recorded for each scenario. Finally, these % BE acceptance values were summarized in the form of histograms for amlodipine, irbesartan, and hydrochlorthiazide (Figure 7). The entire procedure was followed for all batches developed by the R&D and finally the batch referring to the overall highest % probability (concomitantly for the three drugs) was considered the most suitable for use in the pivotal BE study.

## 4. Discussion

The aim of this analysis is to present a new in vitro–in vivo simulation approach that aims to predict the probability of success of a bioequivalence study. This modeling and simulation approach relies on the in vitro release data obtained during the R&D development process and literature information regarding the in vivo pharmacokinetics. Joint simulations of dissolution and in vivo kinetics can be implemented for any number of subjects, any clinical design and further can be repeated for thousands of times using Monte Carlo approaches. A variety of scenarios regarding sample size, sampling scheme, variability is investigated and the probability of success of each one of them is recorded. Finally, an overall assessment of all scenarios is made and a decision can be made whether the tested formulation has substantial chances to show BE and continue with a pivotal BE study. In the opposite situation, the R&D department will have to reformulate the product and after re-test it again with the IVIVS. In case where many different batches are developed, the IVIVS methodology allows selecting the batch with the highest probability of showing BE in the future.

Τhe IVIVS procedure is presented and its predictive ability is assessed through its application during the R&D process of a new generic combination of three drugs (amlodipine 10 mg/irbesartan 300 mg/hydrochlorothiazide 12.5 mg). Using the IVIVS approach, the appropriate batch was selected and the pharmaceutical company used this batch in a pivotal BE study. The IVIVS predictions for BE, were in agreement with the findings of the actual BE study for all three compounds (amlodipine, irbesartan, and hydrochlorthiazide separately). Similarly, successful predictions were obtained in the initial two BE studies where the IVIVS was applied [47].

The actual BE study was successful confirming the findings of the IVIVS procedure. In this study, the entire programming work was implemented in MATLAB^®^, but any other language (e.g., open-source) can be used to perform the IVIVS approach. All parts of the program were validated with other software, as described in the “Methods” section. For example, for the numerical solution of ordinary differential equations the *ode45* function was used and the results of the simulations were compared with those obtained from Simulx (Monolix^®^ 2020R1). Figures S1 and S2 in the Supplementary Materials show the similarity in the results of the IVIVS predictions with the simulations from Simulx^®^. The next steps in the estimation procedure were the calculation of pharmacokinetic parameters (AUC, Cmax, Tmax) and bioequivalence assessment. These tasks were validated by comparing the estimations of the IVIVS with PKanalix^®^ and WinNonlin^®^ findings, which were found the same (Tables S1–S6 in the Supplementary Materials). Additionally, the C-t predictions from the IVIVS (Figure A1, Figure A2 and Figure A3) were in fully accordance with literature reported experimental concentrations of amlodipine [49], irbesartan [50], and hydrochlorthiazide [51,52]. Finally, the use Monte Carlo simulations in bioequivalence was applied in many previous studies of the authors [54].

Since in vitro dissolution data are used to feed the IVIVS tool, some important aspects of the experimental dissolution data should be mentioned. Initially, the discriminatory ability of the in vitro data is important, since the IVIVS predictions depend on them. In case of generic drug development, discriminatory dissolution testing refers to methods exhibiting sensitivity to distinguish changes in the manufacturing process and the formulation. It is generally considered that the typical dissolution conditions (pH of 1.2, 4.5, and 6.8) allow this discrimination [55]. Nevertheless, experience in pharmaceutical industry reveals that in some cases the typical dissolution conditions may fail to unveil the actual dissolution characteristics of the formulations under study. These cases might have an impact on the predictive ability of the IVIVS approach, as in case of any other method. In case of the present example of amlodipine/irbesartan/hydrochlorthiazide, in vitro data came from one-stage dissolution tests using the typical media, volumes etc. Coupling the IVIVS procedure with biopredictive dissolution media can increase its predictive ability. Another method, to allow better predictions of the IVIVS, is to use in vitro data from biphasic dissolution. In this case, a two-phase system is utilized where the simultaneous dissolution and partition into an organic phase is concomitantly assessed [56].

Another situation requiring attention refers to low soluble drugs, where the dissolution data may not come from sink conditions. Thus, the experimental data used to feed the IVIVS system may not be very representative of the in vivo dissolution. However, these situations could be addressed with the use of flow-through dissolution systems, which provide sink conditions much closer to what exists in the GI tract [57]. In addition, as any other methodology utilizing dissolution data, the relationship between the in vivo appearance of drug and dissolution performance is stronger for drugs exhibiting dissolution rate limited absorption; namely, when absorption is much faster than dissolution [58].

A worth mentioning issue is the selection of the dissolution model, which is used during the first step of the IVIVS procedure (Figure 1). For more than 120 years, scientists work on the quantitative analysis of dissolution and several dissolution models have been proposed so far. Initially, Noyes-Whitney (in 1897) and afterwards Nernst-Brunner (in 1904), and Hixson–Crowell (in 1913) provided mechanistic mathematical descriptions of dissolution [59,60]. In addition, the empirical description of the dissolution/release processes was successfully accomplished by the Weibull function or the well-known mathematical equations of the Higuchi model and the Peppas model [61,62,63,64]. In case of the IVIVS, the purpose of using mathematical equations to describe dissolution, only reflects the need of the mathematical description of the data, without trying to investigate or explain the underlying mechanisms. This is due to the fact that the only description of dissolution data is required in order to couple this with the in vivo model. Thus, the simplest model that provides good fitting results is selected in the IVIVS approach. For example, in the cases of the three studied drugs, the first-order model was chosen.

**Figure 8:**
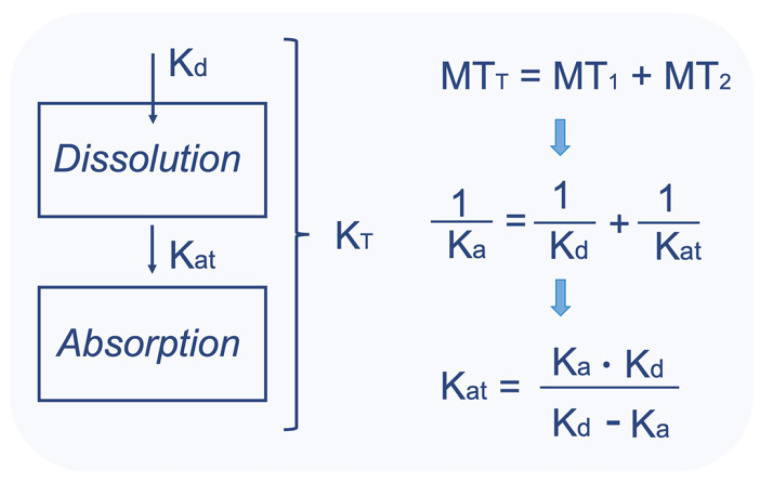
A schematic representation of the two consecutive processes of dissolution and absorption. The mean transit time (MT_T_) of the entire process can be considered equal to the sum of the mean dissolution time (MT_1_) and the mean “true” absorption time (MT_2_) [64]. The “true” absorption rate constant (Kat) can be estimated from the typical absorption rate constant (Ka) and the dissolution rate constant (Kd). First order kinetics are assumed.

Another issue, relevant to the estimations, refers to the distinguishment (in terms of kinetics) of drug dissolution from all other phenomena occurring during drug absorption. In other words, to discriminate the “true” absorption (i.e., permeation) constant from the dissolution rate constant. Traditionally in pharmacokinetics, the estimated rate of absorption refers to the entire process of absorption starting from drug release and ending up with the appearance of drug in plasma, since dissolution is not taken into consideration separately. Thus, the so-estimated constant (i.e., Ka) reflects all kinetic phenomena from drug intake up to the appearance of drug in plasma. In case of the IVIVS methodology, dissolution kinetics is described separately and thus the kinetic part of dissolution, included in Ka, has to be extracted. Drug release (for simplicity it is considered equivalent to drug dissolution) and absorption are consecutive processes, since absorption (i.e., permeation) can only occur after dissolution. The mean transit time (MT_T_) of the entire process can be considered equal to the sum of the mean dissolution time (MT_1_) and the mean true absorption time (MT_2_) (Figure 8) [65]. Thus, solving in terms of MT_2_, the latter can be calculated by subtracting MT_1_ from MT_T_. Assuming first order kinetics for dissolution and absorption, each mean time is the reciprocal of the rate constant (Figure 8). Thus, the “true” absorption rate constant (Kat), namely, the rate constant without the dissolution component can be calculated.

This procedure is applied for the T and R product separately, since different dissolution profiles (i.e., Kd estimates) may exist. Thus, two Kat are estimated: one for the Test formulation (Kat_T) and one for the reference product (Kat_R). Since, the “true” absorption rate constant actually reflects phenomena related only to the active substance (namely, independent from the formulation effects affecting dissolution), then the average of Kat_T and Kat_R is finally estimated and used in the simulations of both compounds. In the IVIVS methodology, the above-mentioned discrimination was made and Kat values were estimated from the literature Ka estimates. Table 5 lists the Kat values along with all other pharmacokinetic values finally used in the simulations.

In the literature, several computational methodologies have appeared and been adopted in the pharmaceutical development. For example, PBPK models are very useful for new drug substances and/or when extrapolating knowledge from animal species to humans in order to predict the in vivo C-t profiles [16,17,18,19]. Other computational methods have been developed for specific purposes, as in the case of drugs delivered via dry powder inhalers for local action in the lungs [20,21]. Additionally, there are software tools simulating several aspects of clinical trials (like sample size, dosage regimens etc.) and intend to predict the probability of success of a future clinical study [18]. And certainly, IVIVC studies, where in the majority of cases are set in a retrospective way, by using deconvolution techniques to correlate the in vivo outcome with the in vitro data in order to support the application of biowaiver [6,66,67]. In other cases, IVIVC tools were used to assess the impact of in vitro variability on in vivo BE criteria, to predict formulation differences in dissolution testing, and to compare convolution and deconvolution methods for the development of IVIVC [40,66,67]. Quite recently an in-silico modeling approach (the “Mechanistic Physiologically-Based Biopharmaceutics Modeling”) was used to predict the in vivo concentrations of ibuprofen through the use of a mechanistic oral absorption model built in the Phoenix WinNonlin^®^ software and coupled with the GastroPlus^®^ simulator [68]. Similarly, aiming at predicting the possibility of bioequivalence for fluconazole capsules, Duque et al., utilized dissolution profiles in order to simulate plasma levels and then evaluate their bioequivalence, using Population Simulator^TM^ in GastroPlus^®^ [41]. This study showed that computer simulations can be an important tool to predict the probability of BE for fluconazole capsules. However, none of these studies are the same with the IVIVS approach. Nevertheless, the IVIVS approach differs from these in many ways. Firstly, the IVIVS procedure does not end up with simulated in vivo profiles or even the assessment of a single simulated BE study, but the entire procedure is repeated for thousands of times in order to get the simulation-based probability of BE acceptance, for each one of the scenarios examined. Combining the results of several scenarios, which mimic different possible situations (e.g., GI transit, sampling schemes etc.) that may occur in vivo, an overall assessment of the possibility of BE success can be obtained.

A brief elaboration on the physicochemical properties (octanol–water partition coefficient (logP), acid dissociation constant (pKa), and molecular weight (MW)) of the three compounds is made in order to relate them with the IVIVS methodology. Amlodipine is a slightly soluble in water base (logP = 3, MW = 567.06, pKa = 9.4) which means that acid media the dissolution can more rapid and in a greater extent, which is in accordance with the experimental dissolution data utilized in this study [69]. Irbesartan is weak acid (pKa1 = 4.08, pKa2 = 4.29) with an aqueous solubility of less than 1mg/mL (logP = 4.5), and MW = 428.5. At low pH values (like those existing in stomach) dissolution is more rapid. Thus, in the IVIVS setting a longer residence in stomach, would result in a greater dissolution and more irbesartan to be absorbed in the small intestine [70]. Hydrochlorthiazide has a water solubility of less than 0.1 mg/mL (MW = 297.7, logP = −0.07) and exerts basic properties (pKa1 = 7.9, pKa2 = 9.2). The latter implies that dissolution is favored at lower pH values, which was in line the experimental dissolution data utilized. Even though that in the conditions of the dissolution trial, the % dissolved reached high values, hydrochlorthiazide is classified as either BCS III (i.e., high solubility/low permeability) or IV (low solubility/low permeability) compound. Application of the IVIVS to low solubility drugs leads to a better discrimination of the in vivo performance [71,72].

Also, more extreme scenarios can be studied and the IVIVS approach can be used as a “stress-test” tool to unveil possible pitfalls of the product under development. In this context, assessing scenarios with different mean transit times for stomach and small intestine can serve as a useful tool to identify differences and magnify their impact on the in vivo kinetics. For example, when an oral solution is compared against an immediate release solid oral formulation (as in case of a hybrid application), the shorter residence in stomach can be investigated in order to unveil the potential more rapid appearance of the oral solution in plasma. The impact of the duration of gastric emptying is more important for rapidly dissolving BCS I drugs (e.g., amlodipine), followed by BCS III and BCS II drugs, while for BCS IV might not be so important. In this study, the application example included three drugs, each one of them belonging to a different BCS class; amlodipine (class I), irbesartan (class II), hydrochlorthiazide (more likely class III, while some others consider it as class IV) [73,74,75].

For regulatory purposes (e.g., grant for a biowaiver), the IVIVS approach is more useful for BCS II and IV compounds, which exert low solubility. However, during the R&D process, the IVIVS can be used for compounds of all BCS classes (including BCS I) in order to select the most appropriate batch among all batches developed, namely, the one leading to the highest probability of declaring bioequivalence in the future BE study. Additionally, the IVIVS can assist the R&D team on the proper design of the release characteristics (i.e., as they are reflected on the dissolution profiles), since the IVIVS translates the in vitro data into the anticipated in vivo performance.

Another point of interest was the selection of scenarios to be assessed from the total number of possible scenarios. In the example of the triple tablet (amlodipine, irbesartan, hydrochlorthiazide) from a pool of 675 scenarios (for each compound), 80 were selected to be analyzed. The scenarios selection should be done in a rational way, which means that the sub-group of scenarios analyzed should be selected to fulfill two independent criteria: (a) being the most probable, (b) refer to most-extreme cases in order to unveil possible differences between the formulations under comparison (as mentioned in the previous paragraph when for example an oral solution is compared to a tablet). In the present analysis, the 80 scenarios referred to independent combinations of: three samples sizes (12, 18, 24), the three sampling schemes, two levels of between-subject variability (5%, 10%), two levels of within-subject variability (10%, 20%), and two transit scenarios (#1 and #2 of Table 1). These combinations lead to 3 × 3 × 2 × 2 × 2 = 72 scenarios. In addition, 8 individual scenarios were tested utilizing specific situations once e.g., one time a scenario with 20% between-subject variability, one scenario with 5% within-subject variability etc. It is worth mentioning that in this analysis there was an additional restriction; all three compounds are included in the same triplet tablet. This implies that in actual conditions one BE will be performed. Thus, our selected scenarios for each compound should fulfill the requirements of all three compounds.

The necessary information to feed the IVIVS tool requires no additional experiments in the R&D, but only suffice the experimental dissolution data and literature information. In some cases, even data from typical (i.e., not population) pharmacokinetic studies, such as Tmax and Cmax, may provide the necessary information to set up the pharmacokinetic model. Of course, the incorporation of more realistic data (e.g., results from a pilot study to estimate pharmacokinetic parameters) increases the predictive ability of the IVIVS. Similarly, when literature population pharmacokinetic data are available, the IVIVS predictions become better.

Several features have already been added in the MATLAB programming code of the IVIVS, such as one- or two-compartment disposition kinetics, enterohepatic recirculation, as well as the existence of parent drug and metabolite and perform BE assessment using both. Any other pharmacokinetic model and dissolution model can be used, simply by setting up the system of ordinary differential equations appropriately.

In the future, the IVIVS approach can be expanded by adding more pharmacokinetic models, coupling it with biopredictive media, simulated in vitro models, and formulation predictive studies [4,14,76,77]. Using the appropriate in vitro data (with adequate discriminatory ability), even predictions for the comparative pharmacological effect can be made, by using joint in vitro—pharmacokinetic—pharmacodynamic simulations. 

**Figure A4:**
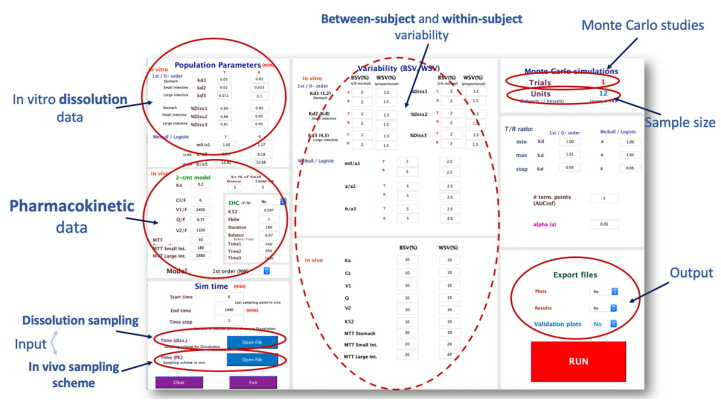
A snapshot of the Matlab^®^ graphical user interface (GUI) used in the in vitro–in vivo simulations of the triple combination tablet (amlodipine, irbesartan, hydrochlorthiazide). Using this simple environment, the user just enters the value in each field, chooses a file, or selects a setting.

Even though the development of such an IVIVS approach requires programming skills, its use can be handled like a black-box by any scientist. For the purposes of performing the analysis of the three drugs, a graphical user interface (GUI) was developed and utilized in all computations (Figure A4 in Appendix B).

## 5. Conclusions

The aim of this study was to present an in vitro–in vivo simulation approach that allows predictions of the probability of success of a bioequivalence study. This IVIVS tool can assist pharmaceutical development by providing guidance to the R&D department on the possibility of BE acceptance of a developed formulation. The most suitable batch can be selected or alternatively the product can be re-formulated, if the IVIVS shows low reduced probability of success. In addition, important aspects of the clinical design of the future pivotal study can be assessed, as for example the appropriate sampling scheme and sample size. Therefore, this guided R&D development of generics can save time and costs.

The IVIVS approach is not a commercial software, but it was developed as an academic research tool. It does not rely on any other software and anyone can implement it in any programming language. In this study, the code was written in MATLAB^®^, but it can also be translated to any language, preferably to an open-source like Python and R, or even can be run on a web-page, like a Python web-app or an R-shiny application.

In this study, the predictability of the IVIVS was evaluated in the development of a new generic triple combination product of amlodipine/irbesartan/hydrochlorothiazide. Based on the IVIVS results, the most appropriate batch of the test product was selected and underwent in BE assessment. The findings of the pivotal BE study confirmed the predictions of the IVIVS.

## Figures and Tables

**Table 1:** Mean transit times (in minutes) at different regions of the gastrointestinal tract (stomach, small intestine, and large intestine). These five combinations of transit times refer to the scenarios utilized in the simulations in this study. In all cases, a total intestinal transit time of 2160 min (i.e., 36 h) is assumed in order to estimate the large intestine transit time.

Location	Mean Transit Time (Minutes)				
	***1***	***2***	***3***	***4***	***5***
**Stomach**	10	20	30	45	60
**Small intestine**	180	200	215	240	265
**Large intestine**	1970	1940	1915	1875	1835

**Table 2:** Sampling schemes investigated in the in vitro–in vivo simulations of amlodipine, irbesartan, hydrochlorothiazide.

Design	Sampling Times (in Minutes)																
***Sparse***	0	10	40	60	90	160	240	480	720	1440							
***Typical***	0	10	20	40	60	90	120	180	240	360	480	720	960	1440			
***Dense***	0	10	20	40	60	80	100	120	150	180	240	360	480	600	720	960	1440

**Table 3:** Scenarios investigated in the in vitro–in vivo simulations of the amlodipine, irbesartan, hydrochlorothiazide combination oral tablet.

**Sample size**	12, 18, 24, 30, 36
**Sampling scheme ^1^**	Typical, Sparse, Dense
**Gastrointestinal transit time ^2^**	Five scenarios
**Between-subject variability**	5%, 10%, 20%
**Within-subject variability**	5%, 10%, 20%

^1^ The sampling schemes utilized in the simulations are quoted in Table 2. ^2^ The scenarios examined are listed in Table 1.

**Table 4:** In vitro dissolution fittings using a first-order model (%Dissolution = m0·Dose(1 − e^−Kd·t^)) for the test formulation (amlodipine/irbesartan/hydrochlorthiazide, 10/300/12.5 mg) at the three buffers (pH: 1.2, 4.5, and 6.8). The terms “m0” and “Kd” refer to the maximum percent of dissolution and the dissolution rate constant, respectively. The average estimate is shown along with the relative standard error (RSE%).

Drug	Buffer	Parameter	Value	RSE%
Amlodipine	*1.2*	**m0**	0.9087	5.28
		**Kd**	1.4954	10.2
	*4.5*	**m0**	0.856	13.28
		**Kd**	0.5734	7.68
	*6.8*	**m0**	0.898	3.18
		**Kd**	0.2886	2.93
Irbesartan	*1.2*	**m0**	0.899	0.865
		**Kd**	0.1726	6.98
	*4.5*	**m0**	0.0234	2.97
		**Kd**	0.1295	4.69
	*6.8*	**m0**	0.7076	3.76
		**Kd**	0.0811	1.26
Hydrochlorthiazide	*1.2*	**m0**	0.9655	6.31
		**Kd**	0.4534	22.78
	*4.5*	**m0**	0.9476	3.28
		**Kd**	0.2358	5.07
	*6.8*	**m0**	0.8874	4.66
		**Kd**	0.2934	3.62

**Table 5:** In vitro and in vivo model parameters either taken from the literature or estimated in the current study. *Key*: Ka, absorption rate constant, F, bioavailability fraction; Cl, clearance; Q, inter-compartmental clearance, V_1_, apparent volume of distribution of the central compartment; V_2_, apparent volume of distribution of the peripheral compartment; Tlag, lag time in absorption; Kd_T and Kd_R, the dissolution rate constant derived from fittings at pH 6.8 for the Test and Reference formulation, respectively; Kat_T and Kat_R, the estimated “true” absorption rate constant for the Test and Reference formulation, respectively; Kat, the “true” absorption rate constant calculated as the average of Kat_T and Kat_R.

Source	Parameter ^4^	Value	Used in Simulations
**Amlodipine**			
**Literature ^1^**	**Ka (min^−1^)**	0.01417	-
	**Cl/F (ml/min)**	370	√
	**V_1_/F (ml)**	1300	√
**In vitro fittings**	**Kd_T (min^−1^)**	0.2886	√
	**Kd_R (min^−1^)**	0.2679	√
**Estimated “true” absorption rate constant**	**Kat_T (min^−1^)**	0.0149	-
	**Kat_R (min^−1^)**	0.01496	-
	**Kat (min^−1^)**	0.01493	√
**Irbesartan**			
**Literature ^2^**	**Tlag (min)**	100.8	√
	**Ka (min^−1^)**	0.00507	-
	**Cl/F (ml/min)**	225	√
	**Q/F (ml/min)**	295	√
	**V_1_/F (ml)**	13,800	√
	**V_2_/F (ml)**	85,800	√
**In vitro fittings**	**Kd_T (min^−1^)**	0.0811	√
	**Kd_R (min^−1^)**	0.0767	√
**Estimated “true” absorption rate constant**	**Kat_T (min^−1^)**	0.00541	-
	**Kat_R (min^−1^)**	0.00544	-
	**Kat (min^−1^)**	0.00543	√
**Hydrochlorthiazide**			
**Literature ^3^**	**Tlag (min)**	24.24	√
	**Ka (min^−1^)**	0.01288	-
	**Cl/F (ml/min)**	575	√
	**Q/F (ml/min)**	423.33	√
	**V_1_/F (ml)**	137,000	√
	**V_2_/F (ml)**	146,000	√
**In vitro fittings**	**Kd_T (min^−1^)**	0.2934	√
	**Kd_R (min^−1^)**	0.2776	√
**Estimated “true” absorption rate constant**	**Kat_T (min^−1^)**	0.01347	-
	**Kat_R (min^−1^)**	0.01351	-
	**Kat (min^−1^)**	0.01349	√

^1^ Obtained from references No. 48 and 49. ^2^ Obtained from reference No. 50. ^3^ Obtained from references No. 51 and 52. ^4^ The “m0” values, utilized in the IVIVS, for each segment of the gastrointestinal tract (stomach fasted, stomach fed, small intestine) are reported in Table 4.

## Data Availability

Data sharing is not applicable to this article.

## Supplementary Materials

The following are available online at https://www.mdpi.com/1996-1944/14/3/555/s1, Figure S1: Comparison of the simulated average C-t profiles between the IVIVS approach (left column) and Simulx^®^ (right column). Simulations refer to amlodipine (a, b), irbesartan (c, d), and hydrochlorthiazide (e, f), Figure S2: A set of simulated amlodipine C-t profiles using the IVIVS approach. The comparative average performance of Test and Reference formulation is shown in (a). The average performance with the sampling points for the Test formulation is depicted in plot (b), while the individual C-t profiles of the virtual subjects receiving the Test formulation are shown in (c). Similarly, the average performance with the sampling points for the Reference product is depicted in plot (d), while the individual C-t profiles of the virtual subjects receiving the Reference formulation are shown in (e). The C,t pairs obtained from the virtual sampling are used in the subsequent steps of the validation procedure for the estimation of the pharmacokinetic parameters (AUC, Cmax) and then for bioequivalence assessment. In this example, 12 subjects were simulated in a 2 × 2 crossover design. Thus, 6 subjects received the Test formulation in the first period of the simulated study and the other 6 subjects in the second period of the study. Similarly, the same applied to the Reference product. Comparison of the plots reveals that the IVIVS leads to the same results as Simulx^®^, Table S1: Selected C-t data of amlodipine (from the sampling scheme) for the 12 virtual subjects (in total for both periods) received the Test formulation and 12 subjects of the Reference product, Table S2: Estimated pharmacokinetic parameters (AUC, Cmax, Tmax) using the IVIVS approach. The C,t data come from Table S1. After the generation of the 12 C-t profiles of Test and Reference formulation, the virtual subjects were randomly assigned into the two treatments, the two sequences, and therefore the two periods of administration of the 2 × 2 crossover design, Table S3: Pharmacokinetic parameters calculated by the PKanalix tool of Monolix^®^ 2020R1. The AUC, Cmax, and Tmax estimates are identical with those calculated by the IVIVS (quoted in Table S2), Table S4: Bioequivalence assessment results derived from the IVIVS. The main bioequivalence estimates are shown: the upper and lower limits of the 90% confidence interval (CI), the geometric mean ratio (GMR), the mean pharmacokinetic parameters for the Test and Reference formulation, and the mean square error from the ANOVA [1,2], Table S5: Bioequivalence assessment results as they are obtained from WinNonlin^®^ v.5.0.1 (Pharsight Corp., Menlo Park, CA). To facilitate the comparison, values in bold refer to the estimates listed in Table S4, Table S6: Additional bioequivalence results, relevant to the ANOVA analysis, from WinNonlin^®^ v.5.0.1 (Pharsight Corp., Menlo Park, CA). To facilitate the comparison with the estimates listed in Table S4, values in referring to the mean square error are annotated in bold type.

## Appendix A

**Table A1:** A representative system of ordinary differential equations, relying on mass balance, incorporated in the IVIVS. The case of first-order dissolution kinetics linked with a two-compartment model (with first-order absorption and elimination from the central compartment) is presented.

Model ^1^	Description ^2^	Initial Conditions
**dy1/dt = − Kd·y1**	Amount of drug undissolved	y1(0) = m0·Dose
**dy2/dt = Kd·y1 − Ka·y2**	Amount of drug dissolved	y2(0) = 0
**dy3/dt = Ka·y2 − K34·y3 + K43** **⋅** **y4 − Kel·y3**	Amount of drug in the central compartment	y3(0) = 0
**dy4/dt = K34·y3 − K43·y4**	Amount of drug in the peripheral compartment	y4(0) = 0

^1^*Key*: Kd, dissolution rate constant; Ka, absorption rate constant; K34, first-order rate constant for the transfer from the central to the peripheral compartment; K43, first-order rate constant for the transfer from the peripheral to the central compartment; Kel, elimination rate constant; m0, the maximum percent of dissolution. ^2^ Drug concentrations in the central and peripheral compartments are calculated from the corresponding amounts by dividing with volume of distribution of the compartment, namely, in case of the central compartment: y3/V1, where V1 is the volume of distribution of the central compartment.

**Table A2:** Additional technical details about the mathematical description used in the in vitro–in vivo simulations (IVIVS) of drug transit through the body ^1^.

**How the IVIVS is implemented**	The IVIVS approach combines: (a) dissolution information (from the in vitro dissolution data), (b) statistics (e.g., starting from the simple typical estimation of bioequivalence according to the guidelines, to the use of probability distribution functions for the random generation of virtual subjects and the application of Monte Carlo simulations), (c) mathematics through the use of systems of differential equations to concomitantly describe the in vitro and in vivo kinetics, and (d) programming in a language to implement all these.
**The mathematical part of the simulations performed in the IVIVS**	1. Drug in the body can be in the following subsequent conditions (parts): (1a) solid drug in the GI (1b) drug dissolved in the GI (1c) drug in the central compartment, and (1d) drug in the peripheral compartment This arrangement derives from the conservation of mass (i.e., amount of drug). An additional compartment of the already eliminated drug could be added, but it would be useless to add one more differential equation to quantify something there is no need to describe it. 2. The GI parts “1a” and “1b” can refer either to fasted stomach with pH 1.2 (or fed stomach with pH 4.5), or small intestine (pH 6.8), or large intestine. 3. The entire situation (i.e., 1a, 1b, 1c, 1d) is described by systems of ordinary differential equations (ODEs). Thus, for our system, there is a need of four differential equations; one for each “part”. 4. In order to express the presence of drug in each different “segment” of the GI (namely, parts “1a” or “1b”), the concept of Mean Transit Time (MTT) is incorporated. 5. Using the MTT, we can define how much time the drug molecules spend within each segment of the GI, namely into segment “1a” or “1b” (since “1c” and “1d” refer to drug inside the body; plasma or tissues). 6. For each segment of the GI (stomach, small intestine, large intestine), different systems of ODEs and parameter values are used: - Drug in stomach (time ≤ MTT_stomach_): The Kd refers to one estimated from the dissolution fittings at pH 1.2. The Ka is usually set to zero, since for most drugs there is no absorption in the stomach. Alternatively, if the drug has some absorption from the stomach, then a fraction of the Ka observed in the small intestine can be used (always according to the literature). - Drug in small intestine (MTT_stomach_ < time ≤ MTT_stomach_ + MTT_SmallIntestine_): The Kd refers to the one estimated from the in vitro fittings at pH 6.8. The Ka is the one (true Ka, Kat) estimated as described in the “Discussion” section and Figure 8. - Drug in large intestine (MTT_stomach_ + MTT_SmallIntestine_ < time ≤ MTT_stomach_ + MTT_SmallIntestine_ + MTT_LargeIntestine_): We don’t actually care for this region, since for most drugs there is no absorption from this area. If absorption needs to be considered (according to the literature information for the drug), then depending on the degree of absorption from large intestine, a fraction of the Kat is set. Since long time has passed (MTTstomach + MTTsmallIntestine), the drug has already dissolved when entering large intestine, so there is no need to describe drug dissolution here. If dissolution needs to be described, then the Kd from small intestine can be used. 7. For example, the system of ODEs described in Table A1, refers to the case when the drug is at the small intestine. The Ka value would be the Kat as presented in the “Discussion” section in order to remove from the Ka reported in the literature all kinetic information due to dissolution. In this example, dissolution is expressed as a first-order process with a kinetic rate constant (Kd) equal to the one estimated from the in vitro fittings at pH 6.8. Other dissolution models can also be used (see ‘Methods’ and ‘Discussion’). 8. Since not all drug molecules enter or leave each GI part concomitantly, variability is added to the MTT. Thus, the residence (or in another view the “transit”) of drug inside the GI has a probabilistic nature according to the probability distribution utilized to add variability to the MTT.
**Some consequences**	- Different formulations have different dissolution profiles and thus, different Kd and m0 values. - Longer residence in the stomach, will result in delayed appearance of drug in the blood, since in stomach no Ka or a low Ka is set in the ODEs. - Possible excipient effect (e.g., potential of laxative effect of PEGs) can be described by setting a shorter MTT in small intestine. Different MTTs can be applied to the Test and Reference formulation. - Fasted vs. fed conditions in stomach are described by: - the different Kd, m0 values that are used to describe the in vivo dissolution (which is based on the values derived from the “in vitro” dissolution fittings from pH 1.2 and 4.5). Different in vitro dissolution profiles would lead to different Kd and m0 estimates. - the longer residence (greater MTT) in stomach in case of fed conditions - Inter-individual variability is added by the typical techniques of random generation of virtual subjects [54], namely, the parameter (e.g., clearance) values are sampled from a probability distribution (log-normal in case of the pharmacokinetic parameters) using the average parameter value reported in the literature and standard deviation any level of between-subject variability we want to define. - Intra-individual variability is added to the previously generated individual values again by random generation through a probability distribution. Two random estimates are generated: the first is applied to the first “occasion” of the simulated individual parameters, the second random estimate is applied to the second “occasion”. Since, it is completely random, the choice of which random error is assigned to the first or second inter-individual estimate, plays no role. - Absorption variability: As mentioned above the true Kat is applied to the absorption from the small intestine. Then depending on the drug (literature information), a fraction of this Ka can be applied to the stomach and/or large intestine.

^1^*Key*: Kd, dissolution rate constant; Ka, absorption rate constant; Kat, true absorption rate constant without the kinetic part of dissolution; m0, the maximum percent of dissolution; MTT, mean transit time; GI, gastrointestinal system; ODEs, ordinary differential equations.
