# Supplementary material for: An In Vitro–In Vivo Simulation Approach for the Prediction of Bioequivalence
Source: Materials (Basel). 2021 Jan 24;14(3):555. doi: 10.3390/ma14030555 (PMC7865526; doi:10.3390/ma14030555)
Supplement: Supplementary file 1 [file materials-14-00555-s001.pdf]

Supplementary Materials: An In Vitro – In Vivo Simulation Approach for the Prediction of Bioequivalence

Marilena Vlachou[1] and Vangelis Karalis[1] *

[1] Department of Pharmacy, School of Health Sciences, National and Kapodistrian University of Athens, Athens, 15784, Greece

* Correspondence: vkaralis@pharm.uoa.gr; Tel.: +30 210 7274267 (V.K.)

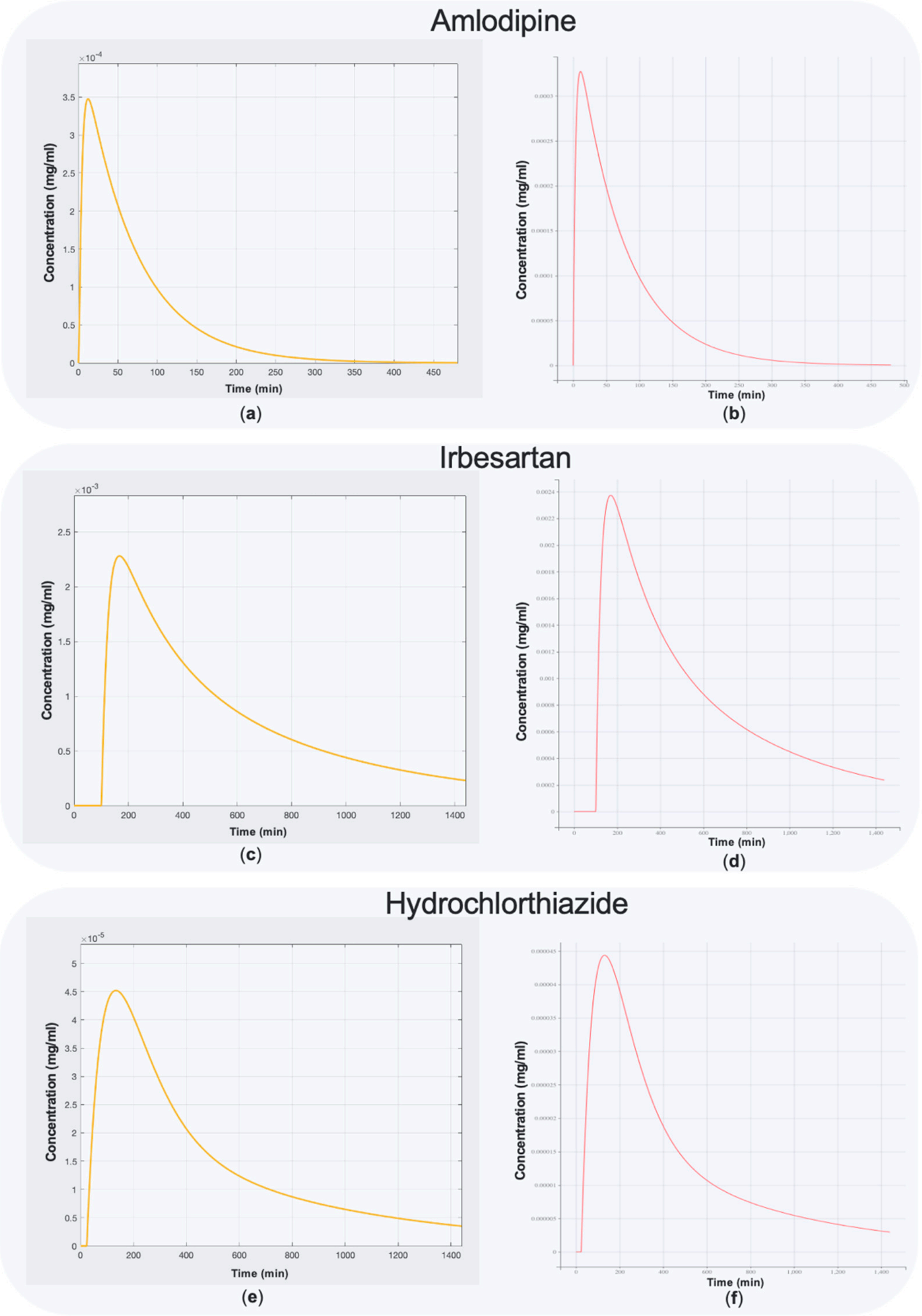

Figure S1. Comparison of the simulated average C-t profiles between the IVIVS approach (left column) and Simulx® (right column). Simulations refer to amlodipine (**a**, **b**), irbesartan (**c**, **d**), and hydrochlorthiazide (**e**, **f**). Comparison of the plots reveals that the IVIVS leads to the same results as Simulx®.

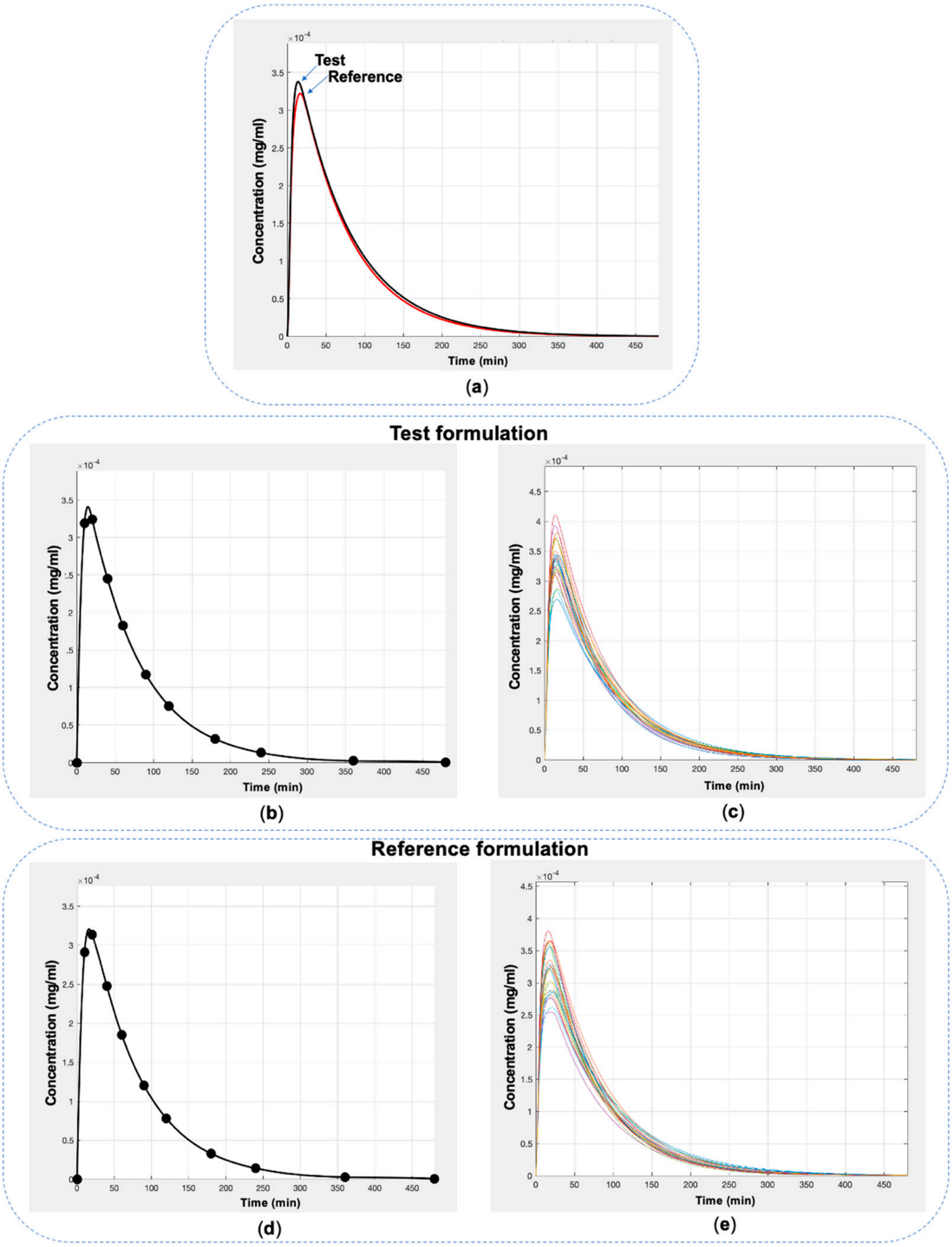

Figure S2. A set of simulated amlodipine C-t profiles using the IVIVS approach. The comparative average performance of Test and Reference formulation is shown in (**a**). The average performance with the sampling points for the Test formulation is depicted in plot (**b**), while the individual C-t profiles of the virtual subjects receiving the Test formulation are shown in (**c**). Similarly, the average performance with the sampling points for the Reference product is depicted in plot (**d**), while the individual C-t profiles of the virtual subjects receiving the Reference formulation are shown in (**e**).

The C,t pairs obtained from the virtual sampling are used in the subsequent steps of the validation procedure for the estimation of the pharmacokinetic parameters (AUC, Cmax) and then for bioequivalence assessment. In this example, 12 subjects were simulated in a 2x2 crossover design. Thus, 6 subjects received the Test formulation in the first period of the simulated study and the other 6 subjects in the second period of the study. Similarly, the same applied to the Reference product.

Table S1. Selected C-t data of amlodipine (from the sampling scheme) for the 12 virtual subjects (in total for both periods) received the Test formulation and 12 subjects of the Reference product.

Test formulation

Time (min)	Subject											
	1	2	3	4	5	6	7	8	9	10	11	12
0	0.00E+00	0.00E+00	0.00E+00	0.00E+00	0.00E+00	0.00E+00	0.00E+00	0.00E+00	0.00E+00	0.00E+00	0.00E+00	0.00E+00
10	3.25E-04	3.55E-04	2.97E-04	3.78E-04	2.64E-04	3.01E-04	2.96E-04	3.09E-04	3.23E-04	3.44E-04	3.04E-04	3.03E-04
20	3.23E-04	3.55E-04	3.13E-04	3.71E-04	2.83E-04	3.23E-04	3.06E-04	3.40E-04	3.19E-04	3.57E-04	2.90E-04	3.12E-04
40	2.46E-04	2.60E-04	2.44E-04	2.65E-04	2.24E-04	2.49E-04	2.33E-04	2.67E-04	2.40E-04	2.73E-04	2.16E-04	2.40E-04
60	1.85E-04	1.88E-04	1.86E-04	1.87E-04	1.72E-04	1.87E-04	1.75E-04	2.03E-04	1.79E-04	2.04E-04	1.60E-04	1.82E-04
90	1.21E-04	1.15E-04	1.24E-04	1.10E-04	1.17E-04	1.21E-04	1.14E-04	1.35E-04	1.15E-04	1.32E-04	1.02E-04	1.20E-04
120	7.91E-05	7.10E-05	8.24E-05	6.54E-05	7.89E-05	7.87E-05	7.38E-05	8.94E-05	7.40E-05	8.58E-05	6.51E-05	7.91E-05
180	3.38E-05	2.68E-05	3.65E-05	2.29E-05	3.61E-05	3.32E-05	3.12E-05	3.93E-05	3.06E-05	3.61E-05	2.64E-05	3.44E-05
240	1.45E-05	1.01E-05	1.62E-05	8.03E-06	1.65E-05	1.40E-05	1.32E-05	1.73E-05	1.26E-05	1.52E-05	1.07E-05	1.50E-05
360	2.64E-06	1.45E-06	3.17E-06	9.87E-07	3.46E-06	2.49E-06	2.34E-06	3.35E-06	2.16E-06	2.68E-06	1.77E-06	2.83E-06
480	4.82E-07	2.07E-07	6.23E-07	1.21E-07	7.24E-07	4.42E-07	4.17E-07	6.47E-07	3.70E-07	4.73E-07	2.93E-07	5.34E-07

Reference formulation

Time (min)	Subject											
	1	2	3	4	5	6	7	8	9	10	11	12
0	0.00E+00	0.00E+00	0.00E+00	0.00E+00	0.00E+00	0.00E+00	0.00E+00	0.00E+00	0.00E+00	0.00E+00	0.00E+00	0.00E+00
10	2.98E-04	3.31E-04	2.94E-04	3.01E-04	2.73E-04	3.00E-04	2.55E-04	2.53E-04	2.66E-04	3.43E-04	2.44E-04	2.88E-04
20	3.18E-04	3.65E-04	3.19E-04	3.25E-04	3.01E-04	3.29E-04	2.76E-04	2.84E-04	2.80E-04	3.61E-04	2.54E-04	3.24E-04
40	2.43E-04	2.90E-04	2.51E-04	2.49E-04	2.43E-04	2.59E-04	2.24E-04	2.42E-04	2.25E-04	2.70E-04	2.04E-04	2.63E-04
60	1.80E-04	2.18E-04	1.88E-04	1.83E-04	1.82E-04	1.93E-04	1.72E-04	1.89E-04	1.70E-04	1.96E-04	1.53E-04	2.01E-04
90	1.16E-04	1.42E-04	1.21E-04	1.16E-04	1.17E-04	1.25E-04	1.17E-04	1.30E-04	1.11E-04	1.22E-04	9.86E-05	1.33E-04
120	7.40E-05	9.25E-05	7.78E-05	7.34E-05	7.53E-05	8.02E-05	7.88E-05	8.98E-05	7.27E-05	7.56E-05	6.37E-05	8.87E-05
180	3.03E-05	3.93E-05	3.23E-05	2.94E-05	3.13E-05	3.33E-05	3.61E-05	4.26E-05	3.11E-05	2.91E-05	2.66E-05	3.92E-05
240	1.24E-05	1.67E-05	1.34E-05	1.18E-05	1.30E-05	1.38E-05	1.65E-05	2.02E-05	1.33E-05	1.12E-05	1.11E-05	1.73E-05
360	2.09E-06	3.01E-06	2.31E-06	1.89E-06	2.23E-06	2.37E-06	3.45E-06	4.56E-06	2.45E-06	1.66E-06	1.93E-06	3.39E-06
480	3.50E-07	5.45E-07	3.98E-07	3.03E-07	3.85E-07	4.08E-07	7.22E-07	1.03E-06	4.50E-07	2.47E-07	3.36E-07	6.61E-07

Table S2. Estimated pharmacokinetic parameters (AUC, Cmax, Tmax) using the IVIVS approach. The C,t data come from Table S1. After the generation of the 12 C-t profiles of Test and Reference formulation, the virtual subjects were randomly assigned into the two treatments, the two sequences, and therefore the two periods of administration of the 2x2 crossover design.

Subject	Sequence	Period	Formulation	AUC	Cmax	Tmax
1	1	1	1	0.02712	0.00032	20
2	2	2	1	0.03270	0.00036	20
3	2	2	1	0.02802	0.00032	20
4	1	1	1	0.02730	0.00032	20
5	2	2	1	0.02688	0.00030	20
6	1	1	1	0.02883	0.00033	20
7	2	2	1	0.02663	0.00028	20
8	1	1	1	0.02933	0.00028	20
9	2	2	1	0.02559	0.00028	20
10	1	1	1	0.02917	0.00036	20
11	1	1	1	0.02282	0.00025	20
12	2	2	1	0.03039	0.00032	20
1	1	2	2	0.02850	0.00032	10
2	2	1	2	0.02813	0.00036	10
3	2	1	2	0.02868	0.00031	20
4	1	2	2	0.02778	0.00038	10
5	2	1	2	0.02683	0.00028	20
6	1	2	2	0.02825	0.00032	20
7	2	1	2	0.02669	0.00031	20
8	1	2	2	0.03102	0.00034	20
9	2	1	2	0.02734	0.00032	10
10	1	2	2	0.03107	0.00036	20
11	1	2	2	0.02450	0.00030	10
12	2	1	2	0.02802	0.00031	20

Table S3. Pharmacokinetic parameters calculated by the PKanalix tool of Monolix® 2020R1. The AUC, Cmax, and Tmax estimates are identical with those calculated by the IVIVS (quoted in Table S2).

id	AUCINF_obs	AUClast	Cl_obs	Clast	Cmax	HL_Lambda_z	Lambda_z	Tlast	Tmax	Treat
1	0.027	**0.027**	368.33	3.5E-07	**0.00032**	46.69	0.015	480	**20**	1
2	0.033	**0.033**	305.34	5.4E-07	**0.00037**	48.73	0.014	480	**20**	1
3	0.028	**0.028**	356.53	4E-07	**0.00032**	47.43	0.015	480	**20**	1
4	0.027	**0.027**	366.11	3E-07	**0.00033**	45.54	0.015	480	**20**	1
5	0.027	**0.027**	371.49	3.9E-07	**0.0003**	47.28	0.015	480	**20**	1
6	0.029	**0.029**	346.31	4.1E-07	**0.00033**	47.39	0.015	480	**20**	1
7	0.027	**0.027**	374.67	7.2E-07	**0.00028**	53.33	0.013	480	**20**	1
8	0.029	**0.029**	340.58	0.000001	**0.00028**	55.85	0.012	480	**20**	1
9	0.026	**0.026**	390.21	4.5E-07	**0.00028**	49.24	0.014	480	**20**	1
10	0.029	**0.029**	342.58	2.5E-07	**0.00036**	43.64	0.016	480	**20**	1
11	0.023	**0.023**	437.65	3.4E-07	**0.00025**	47.58	0.015	480	**20**	1
12	0.03	**0.03**	328.83	6.6E-07	**0.00032**	51.14	0.014	480	**20**	1
13	0.029	**0.029**	350.36	4.8E-07	**0.00033**	48.95	0.014	480	**10**	2
14	0.028	**0.028**	355.41	2.1E-07	**0.00036**	42.77	0.016	480	**10**	2
15	0.029	**0.029**	348.01	6.2E-07	**0.00031**	51.14	0.014	480	**20**	2
16	0.028	**0.028**	359.81	1.2E-07	**0.00038**	39.68	0.017	480	**10**	2
17	0.027	**0.027**	371.87	7.2E-07	**0.00028**	53.27	0.013	480	**20**	2
18	0.028	**0.028**	353.49	4.4E-07	**0.00032**	48.23	0.014	480	**20**	2
19	0.027	**0.027**	374.24	4.2E-07	**0.00031**	48.25	0.014	480	**20**	2
20	0.031	**0.031**	321.74	6.5E-07	**0.00034**	50.74	0.014	480	**20**	2
21	0.027	**0.027**	365.68	3.7E-07	**0.00032**	47.12	0.015	480	**10**	2
22	0.031	**0.031**	321.62	4.7E-07	**0.00036**	48.04	0.014	480	**20**	2
23	0.024	**0.024**	408.23	2.9E-07	**0.0003**	46.19	0.015	480	**10**	2
24	0.028	**0.028**	356.59	5.3E-07	**0.00031**	49.98	0.014	480	**20**	2

Table S4. Bioequivalence assessment results derived from the IVIVS. The main bioequivalence estimates are shown: the upper and lower limits of the 90% confidence interval (CI), the geometric mean ratio (GMR), the mean pharmacokinetic parameters for the Test and Reference formulation, and the mean square error from the ANOVA [1,2].

**Bioequivalence measure**	**Parameter**	**Value**
AUC	Lower 90% CI	0.97645
	Upper 90% CI	1.040459
	GMR	1.007947
	Mean_Test	0.02801523
	Mean_Reference	0.02779435
	Mean square error	0.001841
Cmax	Lower 90% CI	1.00207
	Lower 90% CI	1.10664
	GMR	1.053058
	Mean_Test	0.000324693
	Mean_Reference	0.000308334
	Mean square error	0.004099

Table S5. Bioequivalence assessment results as they are obtained from WinNonlin® v.5.0.1 (Pharsight Corp., Menlo Park, CA). To facilitate the comparison, values in bold refer to the estimates listed in Table S4.

Dependent	Ln(auc)	Ln(cmax)
**FormRef**	1	1
**RefLSM**	-3.5829	-8.08
**RefLSM_SE**	0.0232	0.0279
**RefGeoLSM**	**0.0278**	**0.0003**
**Test**	2	2
**TestLSM**	-3.575	-8.0296
**TestLSM_SE**	0.0232	0.0279
**TestGeoLSM**	**0.028**	**0.0003**
**Difference**	0.0079	0.0504
**Diff_SE**	0.0175	0.0258
**Diff_DF**	10	10
**Ratio[%Ref]**	**100.79**	**105.17**
**CI_80_Lower**	98.4	101.51
**CI_80_Upper**	103.24	108.96
**WL_80_Lower**	97.36	92.41
**WL_80_Upper**	102.64	107.59
**CI_90_Lower**	**97.64**	**100.36**
**CI_90_Upper**	**104.04**	**110.21**
**WL_90_Lower**	96.54	91.04
**WL_90_Upper**	103.46	108.96
**CI_95_Lower**	96.93	99.29
**CI_95_Upper**	104.8	111.4
**WL_95_Lower**	95.77	89.79
**WL_95_Upper**	104.23	110.21

Table S6. Additional bioequivalence results, relevant to the ANOVA analysis, from WinNonlin® v.5.0.1 (Pharsight Corp., Menlo Park, CA). To facilitate the comparison with the estimates listed in Table S4, values in referring to the mean square error are annotated in bold type.

Dependent	Hypothesis	DF	SS	MS	F_stat	P_value
Ln(auc)	seq	1	0	0	0	0.9621
Ln(auc)	seq*sub	10	0.1111	0.0111	6.03	0.0044
Ln(auc)	form	1	0.0004	0.0004	0.2	0.6627
Ln(auc)	period	1	0.006	0.006	3.24	0.1019
Ln(auc)	Error	10	0.0184	**0.0018**		
Ln(cmax)	seq	1	0.0071	0.0071	0.49	0.5017
Ln(cmax)	seq*sub	10	0.1467	0.0147	3.67	0.0261
Ln(cmax)	form	1	0.0152	0.0152	3.81	0.0793
Ln(cmax)	period	1	0.0067	0.0067	1.68	0.2246
Ln(cmax)	Error	10	0.04	**0.004**		
